# Discovery of Novel Hsp90 C-Terminal Inhibitors Using 3D-Pharmacophores Derived from Molecular Dynamics Simulations

**DOI:** 10.3390/ijms21186898

**Published:** 2020-09-20

**Authors:** Tihomir Tomašič, Martina Durcik, Bradley M. Keegan, Darja Gramec Skledar, Živa Zajec, Brian S. J. Blagg, Sharon D. Bryant

**Affiliations:** 1 Faculty of Pharmacy, University of Ljubljana, Aškerčeva 7, 1000 Ljubljana, Slovenia; martina.durcik@ffa.uni-lj.si (M.D.); darja.gramec-skledar@ffa.uni-lj.si (D.G.S.); ziva.zajec@ffa.uni-lj.si (Ž.Z.); 2 Department of Chemistry and Biochemistry, The University of Notre Dame, 305 McCourtney Hall, Notre Dame, IN 46556, USA; bkeegan@nd.edu (B.M.K.); bblagg@nd.edu (B.S.J.B.); 3 Inte:Ligand Softwareentwicklungs- und Consulting GmbH, Mariahilferstrasse 74B, 1070 Vienna, Austria; bryant@inteligand.com

**Keywords:** allosteric, cancer, Hsp90, inhibitor, molecular dynamics, pharmacophores, virtual screening

## Abstract

Hsp90 C-terminal domain (CTD) inhibitors are promising novel agents for cancer treatment, as they do not induce the heat shock response associated with Hsp90 N-terminal inhibitors. One challenge associated with CTD inhibitors is the lack of a co-crystallized complex, requiring the use of predicted allosteric apo pocket, limiting structure-based (SB) design approaches. To address this, a unique approach that enables the derivation and analysis of interactions between ligands and proteins from molecular dynamics (MD) trajectories was used to derive pharmacophore models for virtual screening (VS) and identify suitable binding sites for SB design. Furthermore, ligand-based (LB) pharmacophores were developed using a set of CTD inhibitors to compare VS performance with the MD derived models. Virtual hits identified by VS with both SB and LB models were tested for antiproliferative activity. Compounds **9** and **11** displayed antiproliferative activities in MCF-7 and Hep G2 cancer cell lines. Compound **11** inhibited Hsp90-dependent refolding of denatured luciferase and induced the degradation of Hsp90 clients without the concomitant induction of Hsp70 levels. Furthermore, compound **11** offers a unique scaffold that is promising for the further synthetic optimization and development of molecules needed for the evaluation of the Hsp90 CTD as a target for the development of anticancer drugs.

## 1. Introduction

Cancer remains one of the leading causes of disease, mortality and economic loss worldwide [1]. While there have been considerable improvements in treatment and survival times for patients suffering from a number of different types of cancer, there is still an urgent need to identify novel molecular targets, curative therapies and anticancer agents with improved efficacy and reduced adverse outcomes [2,3]. Hsp90 is a highly conserved molecular chaperone that is responsible for the folding, activation and maturation of more than 300 client proteins, including protein kinases, E3-ligases and transcription factors [4,5]. Since these Hsp90 clients are involved and play important roles in cancer, Hsp90 has been recognized as a promising target for development of anticancer drugs [6]. Hsp90 is a homodimer composed of the N-terminal domain (NTD) with ATPase activity, the middle domain important for client and co-chaperone interactions, and the C-terminal domain (CTD), which mediates dimerization [5,7]. All Hsp90 inhibitors that have entered into clinical trials for the treatment of cancer bind to the ATP-binding site at Hsp90 NTD, and have displayed significant limitations, including unwanted side effects related to the induction of the heat shock response, as well as limited efficacy and dose-limiting toxicities, such as hepatotoxicity [8,9,10]. One of the major drawbacks associated with ATP-competitive Hsp90 NTD inhibition is the induction of the pro-survival heat shock response, which occurs at the same concentration that is required to outcompete the abundant ATP present in the cellular environment and to induce client degradation. The heat shock response results in increased levels of Hsp90 and anti-apoptotic proteins, such as heat shock factor 1 (HSF-1), Hsp27, and Hsp70, which initiate anti-apoptotic cascades and promote drug resistance [11]. Consequently, alternative mechanisms, such as selective Hsp90β inhibition [12], allosteric Hsp90 CTD inhibition [13], covalent Hsp90 inhibition [14], and/or targeting protein-protein interactions between Hsp90 and its co-chaperones [15], are promising new approaches being pursued [16].

**Figure 1:**
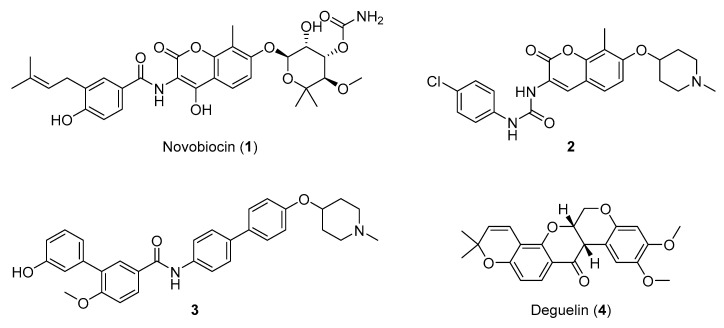
Representative Hsp90 C-terminal domain inhibitors. First Hsp90 C-terminal domain (CTD) inhibitor **1** (IC_50_ ~700 μM SKBr3 cancer cells) [17,21], synthetic novobiocin derivatives **2** and **3** (IC_50_ < 1 μM SKBr3 cancer cells) [22,23] and deguelin (**4**), a flavin natural product.

The Hsp90 CTD contains a secondary nucleotide-binding site, which only becomes available after ATP binds to the Hsp90 NTD [17,18,19]. The aminocoumarin antibiotic novobiocin (Figure 1, compound **1**) was the first compound shown to bind the Hsp90 CTD and induce the proteasomal degradation of oncogenic clients, such as Raf-1, v-Src, Her2 and mutated p53, by inhibiting their correct folding [17,20,21]. In contrast to Hsp90 NTD inhibitors, novobiocin did not induce the heat shock response after the treatment of cancer cells at concentrations needed for client protein degradation [17]. Since its discovery, several focused libraries have been synthesized to improve efficacy and explore the structure-activity relationships of CTD inhibitors. For example, replacement of the noviose sugar with ionizable amines (Figure 1, compounds **2** and **3**), resulted in significant improvements in activity against various cancer cell lines [22,23]. In addition, the coumarin core of **2** could be replaced with biphenyl substituents (**3**) without losses of efficacy. Other natural products (e.g., epigallocatechin 3-gallate, coumermycin A1 and deguelin (Figure 1, compound **4**)) also exhibited antiproliferative activity through Hsp90 CTD inhibition [13].

Though the efficacies of natural product analogues were much improved compared to the parent compounds, so far, none have been co-crystallized with the Hsp90 CTD, limiting structure-based design approaches. In fact, whereas the Hsp90 NTD ATP-binding pocket is well described and several crystal structures with ATP, and inhibitors have been published, the exact location of the allosteric pocket in Hsp90 CTD remains unknown. Several approaches to predict the Hsp90 CTD binding site using both experimental and molecular modeling methods have been reported. Matts and coworkers studied the potential Hsp90 CTD allosteric binding site by protease fingerprinting and photoaffinity labeling utilizing LC–MS/MS. From these experiments, they built a model of Hsp90 in the open conformation based on homology modeling and small angle X-ray scattering (SAXS) of the open structure of HtpG [24]. In another study, molecular dynamics (MD) simulations and signal propagation analyses were used to identify an allosteric binding site in the CTD of yeast Hsp90. In both cases, putative binding poses of novobiocin and some analogues were predicted by molecular docking [25]. More recently, Colombo and coworkers applied funnel metadynamics to probe the binding of an Hsp90 activator in the CTD allosteric binding site [26]. They reported three minima corresponding to different binding modes. One was identical to a site that they used for a previous molecular docking study [26,27]. While these studies provide interesting insights into the conformational flexibility of the CTD and binding mode of novobiocin and some analogues, the lack of X-ray data to confirm a mode of action limits structure-based design approaches.

To further investigate a molecular basis for CTD inhibition with structure-based methodology and identify a new structural class of inhibitors, we utilized the full-length Hsp90β cryoEM structure (PDB Code: 5FWK) with bound ATP [28] for CTD binding site prediction, followed by MD simulations of novobiocin and compound **2**. In contrast to previous studies, we incorporated a novel method to analyze and extract 3D-chemical feature interactions between the ligands and CTD binding sites across the MD trajectory in the form of an ensemble of unique 3D-pharmacophore models. To further assess the MD-derived structure-based (SB) pharmacophores, we developed ligand-based (LB) pharmacophore models based on 13 active novobiocin analogues to compare the resulting chemical features. Both the SB-MD-derived and LB-pharmacophore models were used for virtual screening (VS) of commercially available molecules. Prioritized hits were evaluated for antiproliferative activity in liver and breast cancer cell lines, as well as luciferase refolding assays to assess Hsp90 inhibition and Western blots to check for the client protein degradation and the induction of heat shock response. Herein, we describe our approach for identifying a novel Hsp90 CTD inhibitor using MD-derived SB-pharmacophore models.

## 2. Results and Discussion

### 2.1. Ligand-Based Pharmacophore Modeling

The goals of the LB modeling study were threefold: (1) To address the important question of which chemical features a set of highly active Hsp90 CTD inhibitors have in common in the 3D-space; (2) To utilize the LB model as a query to identify molecules with similar interaction patterns, and unique scaffolds via VS of commercially available compounds; and (3) To compare the features of LB models based on a set of known Hsp90 CTD inhibitors, to SB models derived from MD simulations, to make sense of interactions derived from a predicted allosteric binding site.

**Figure 2:**
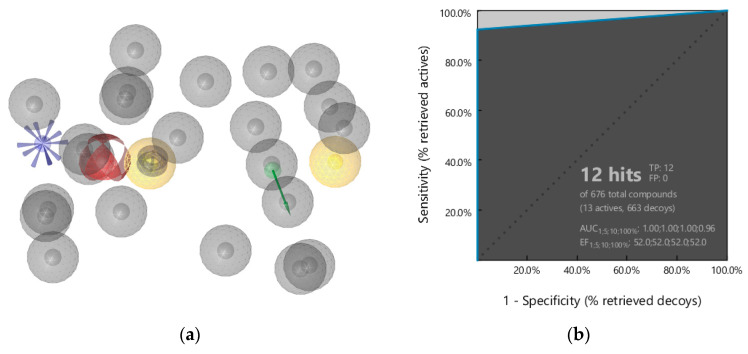
(**a**) Ligand-based pharmacophore model for Hsp90 CTD inhibitors. Common chemical features of the aligned molecules include 2 hydrophobic (yellow spheres), one with an aromatic feature (blue disc), a hydrogen bond (H-bond) donor (green arrow) indicating a defined direction for H-bonding, H-bond acceptor (red sphere), positive ionizable (blue star), and exclusion volumes (grey spheres) that define restricted regions based on the shape of the aligned molecules. (**b**) Resulting receiver operating (ROC) plot (curve shown in blue) from virtually screening 676 compounds (13 Hsp90 CTD actives and 663 generated decoys) with the ligand-based pharmacophore model. TP = true positives; FP = false positives; AUC = area under the curve; EF = enrichment factor.

A set of 13 Hsp90 CTD inhibitors containing both coumarin and biphenyl scaffolds (IC_50_ values ranging from 0.13 μM–0.5 μM against SKBr-3 breast cancer cells) [22,23] were selected for LB modeling (Supplementary Table S1). The selected derivatives exhibited more than 1000-fold improved activities compared to novobiocin (IC_50_ ~700 μM), the first Hsp90 CTD inhibitor. In the absence of bioactive conformations, up to 200 conformations were calculated for each inhibitor and multiple alignments based on the chemical features in 3D-space of all conformations were accomplished using LigandScout 4.3 Expert [29]. Ten 3D-pharmacophore models with the best alignments were generated and ranked with pharmacophore alignment scores. Exclusion volume (EV) features (grey spheres) were automatically added to each model based on the shape of the ensemble of aligned molecules (Figure 2a). EVs represent restricted space important for increasing the selectivity of a model for virtual screening campaigns.

**Figure 3:**
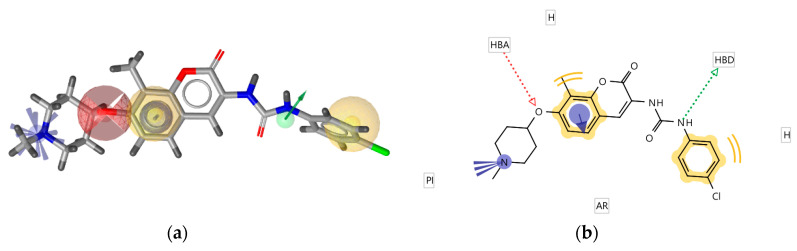
Alignment of compound **2** with the 3D-ligand-based pharmacophore model selected for virtual screening, (**a**) 3D-model and (**b**) 2D-projection. The model was derived using LigandScout 4.3 [29]. Exclusion volumes not displayed.

The LB-pharmacophore models were evaluated, considering the resulting features and receiver operating characteristic (ROC) curves generated by screening libraries of actives (13 novobiocin derivatives) and decoy (663) compounds. The LB model prioritized for the virtual screening is shown in Figure 2a and a ROC curve is shown in Figure 2b. Figure 3a displays the alignment of compound **2** with the LB model and exemplifies the chemical features that the 13 potent CTD inhibitors share in the 3D-space.

On one side of the model, a hydrogen bond (H-bond) donor (green vector) with defined direction is present, due to the alignment of the amide nitrogen atoms in all 13 active compounds. The nearby hydrophobic feature (yellow sphere) is common to 12 of the 13 derivatives. Compound **S12** (Supplementary Table S1) contains a triazole in that position of the alignment that cannot match the hydrophobic feature. Though the selected model does not retrieve all 13 actives, it was prioritized due to the presence of the common positive ionizable feature (blue star). The feature is important, as previous studies reported that the replacement of the noviose sugar in novobiocin with ionizable amines resulted in significant increases in efficacy [22,23]. Other common features included hydrophobic-aromatic (yellow sphere and blue disc), resulting from the common alignment of aromatic rings and a nearby H-bond acceptor (red sphere), representing the ether oxygen present in all of the 13 Hsp90 CTD inhibitors (Figure 2a and Figure 3, Supplementary Table S1).

A ROC curve (Figure 2b) was generated for comparing the MD-derived SB models using the same active and decoy datasets. The LB model exhibited a desirable sensitivity and selectivity with a high enrichment factor (EF = 52) and strong area under the curve (AUC = 1.0 at 1%, 5%, 10% and 0.96 at 100% of the library), as it retrieved almost all of the compounds in the desired active space and none of the decoys (Figure 2b). In addition, it displayed a desirable overall low hit rate (1.8%); deemed suitable for virtual screening large commercially available compound libraries, with the aim of identifying a low number of hits that match the chemical features in the 3D-space of optimized novobiocin analogues but with novel scaffolds. Furthermore, an additional test set of 15 Hsp90 N-terminal domain inhibitors extracted from resolved X-ray structures reported in the Protein Data Bank was screened with the prioritized model, and none of the NTD inhibitors were retrieved.

### 2.2. Identification of the Hsp90 Allosteric Binding Site at C-Terminal Domain

**Figure 4:**
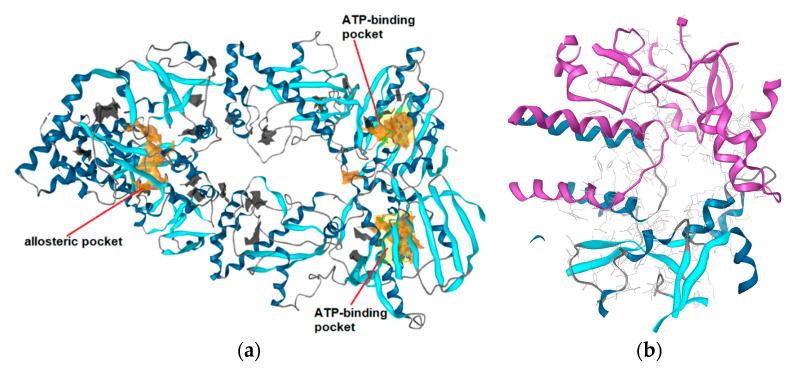
(**a**) Identification of putative binding pockets in Hsp90β cryoEM structure (PDB Code: 5FWK). The orange isosurfaces represent prioritized pockets identified by LigandScout 4.3. Two predicted pockets correspond to the ATP-binding sites in N-terminal domain of each Hsp90 monomer. A third large orange isosurface represents the allosteric pocket at the interface of two C-terminal domains. (**b**) Predicted allosteric pocket in the Hsp90 CTD at the interface of monomer A (magenta) and monomer B (blue) that was prioritized for docking, molecular dynamics and structure-based (SB)-pharmacophore modeling studies.

The full-length Hsp90β cryoEM structure with bound ATP (PDB Code: 5FWK) [28] was used for CTD binding site prediction. The pocket finder algorithm in LigandScout 4.3 identified several binding pockets in the structure, as shown in Figure 4a. The orange isosurfaces represent pockets that were recommended by the program as druggable. The ATP binding sites were correctly identified at the Hsp90 NTD of each monomer, while a third large binding pocket was prioritized at the interface between two Hsp90 CTDs. The third allosteric pocket (Figure 4b) was selected for docking studies, MD simulations with novobiocin and compound **2**, and the generation of SB pharmacophore models. It was delineated by residues 489, 603–613, 660–673 and 676 (monomer A), and residues 489–493, 515, 516, 601–609 and 663–677 (monomer B), and was similar to an allosteric binding site identified previously in yeast Hsp82 [25].

### 2.3. Binding Modes of Novobiocin and Compound **2** in the Hsp90 CTD Allosteric Pocket

**Figure 5:**
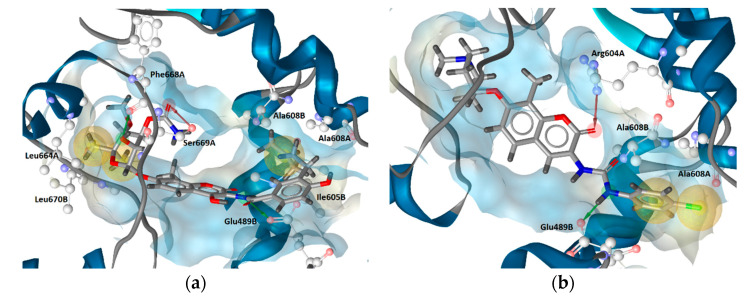
Prioritized docked poses of (**a**) novobiocin and (**b**) compound **2** in a predicted putative allosteric pocket in the Hsp90β C-terminal domain derived from the cryo-EM structure (PDB Code: 5FWK). For clarity, only amino acids interacting with respective compounds are shown. Hydrophobic features are shown as yellow spheres, H-bond donor as green arrow and H-bond acceptor as red arrow indicating a defined direction for H-bonding. The prioritized poses were used as starting points for molecular dynamics-based (MD) simulations.

To investigate potential interactions between CTD inhibitors and the predicted allosteric binding site, novobiocin and compound **2** were docked into the apo pocket, and each subjected to 20 ns MD simulations. Novobiocin, though only moderately active compared to optimized derivatives, was selected for comparing results to previously reported studies on its predicted binding mode. All of the 13 CTD inhibitors with high antiproliferative activities used for LB modeling were docked into the predicted allosteric site using AutoDock Vina, as implemented in LigandScout 4.3. Compound **2** was selected for the MD simulation and SB-pharmacophore modeling based on the high antiproliferative activity and low molecular weight (Supplementary Table S1). The docked poses prioritized for the MD simulations of both compounds are shown in Figure 5.

As expected, docking of the more bulky novobiocin into the apo pocket encountered some steric clashes and fewer reasonable poses in contrast to compound **2**. However, similar binding modes could be identified that included hydrophobic interactions with Ala608A and Ala608B, and H-bonding between Glu489B and the amide NH of novobiocin and the urea NHs of compound **2**. H-bond interactions with Glu residues in both regions were reported in other docking studies as well, though not in the same positions [25,26,27]. The observed H-bonding with the amide/urea groups was promising, since the LB model contained a directional H-bond donor feature based on alignment of amide and urea functional groups of the potent Hsp90 CTD inhibitors (Figure 3). Moreover, both compounds also exhibited unique interactions. For example, compound **2** displayed H-bonding with Arg604A (Figure 5b) and the coumarin carbonyl oxygen, whereas the coumarin moiety of the novobiocin docked pose did not display any interactions. However, the novobiocin docked pose exhibited H-bonding with the noviose 2′-hydroxyl group and Phe668A backbone carbonyl, as well as with the noviose carbamoyl moiety and the Ser669A and hydrophobic interactions with Ile605B, Leu664A and Leu670B (Figure 5a). In contrast, binding partners for the important tertiary amine of compound **2** were not observed in the docked pose, nor were interactions with the ether oxygen.

To further address potential issues associated with a poorly adapted apo pocket, both prioritized poses were used as starting points for MD simulations to explore other interactions and develop SB-models for VS.

### 2.4. Molecular Dynamics Analysis: Interactions and Binding Modes in the Allosteric Hsp90 CTD Binding Site

**Figure 6:**
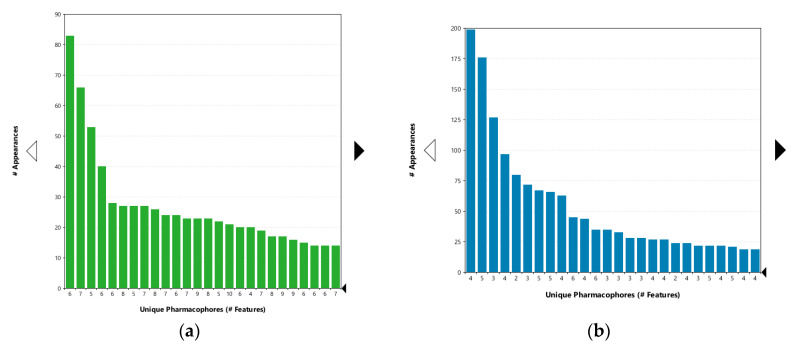
Plot of the most frequently appearing unique pharmacophore models derived from MD simulations of the Hsp90 CTD domain in complex with (**a**) novobiocin (green) and (**b**) compound **2** (blue). The *x*-axis shows unique models and the number of interaction features observed during the MD simulation. The numbers below the bar indicate the number of interaction features in the pharmacophore models. The *y*-axis shows the frequency of appearance of the models. The most frequently occurring model in the novobiocin simulation had 6 interaction features, while the most frequently occurring model for compound **2** had 4 interaction features. The features and binding partners are displayed in Figure 7.

**Figure 7:**
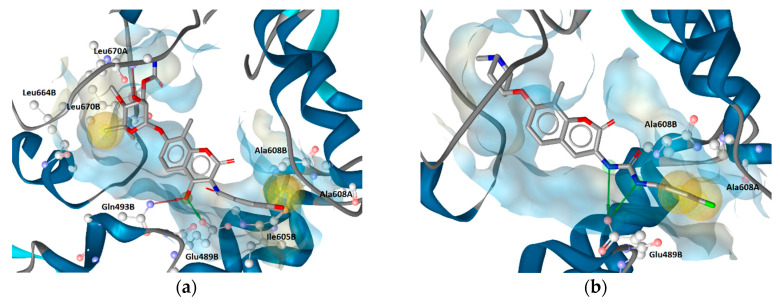
Chemical feature-based pharmacophore features of the most frequently occurring interactions and binding modes of (**a**) novobiocin (yellow) and (**b**) compound **2** derived from 20 ns MD simulations with the Hsp90β C-terminal domain (5FWK). For clarity, only amino acids interacting with the respective ligands are shown. A hydrophobic feature is shown as yellow sphere, H-bond donor as green arrow and H-bond acceptor as red arrow, indicating a defined direction for H-bonding.

The interaction features between the ligands and the Hsp90 CTD pocket were analyzed using MD analysis tools in LigandScout 4.3 Expert. Figure 6 shows plots of the unique and most frequently appearing models, the total number of interaction features they contain (*x*-axis) and the frequency (number of appearances—*y*-axis) in which they occurred during the 20 ns simulation for novobiocin (Figure 6a) and compound **2** (Figure 6b). In the case of novobiocin, the most frequently occurring models had 6 interaction features and appeared more than 80 times during the simulation. Figure 7a displays the binding mode and pharmacophore feature interactions. In comparison to the docked pose of novobiocin before MD (Figure 5a), the amino acid side chains had adjusted to the presence of a large ligand, and important H-bonds between the coumarin structure and Glu489B and Gln493B were observed. In addition, hydrophobic contacts were formed with Ile605B, Ala608A, Ala608B, Leu664B, Leu670A and Leu670B. Additional interactions were observed in the second and fifth most frequently appearing, each containing seven and eight interactions, respectively.

In the case of compound **2**, the most frequently appearing interaction models (>185 times) during the MD simulation had four interaction features, as shown in Figure 7b. Compared to the docked pose (Figure 5b) the H-bond interaction between coumarin and Arg604A was lost. Both of the urea NH groups formed hydrogen bonds with Glu489B, while the hydrophobic interactions formed with Ala608A and Ala608B were the same. A different binding mode of analogue **S2** (Supplementary Table S1) based on docking and MD simulations was reported previously [23]. In particular, the tertiary amine was bound in proximity to Glu477A (yeast Hsp82 numbering, PDB Code 2CG9), where an ionic interaction was noted as well as several hydrophobic interactions with the dimethylphenyl moiety. Although the coumarin ring and urea moiety of **S2** were bound close to Glu477B, the coumarin was flipped compared to the binding modes of compound **2** reported herein. Further experimental studies are needed to elucidate the actual binding mode of compound **2** and its analogues in the Hsp90 CTD binding site.

**Figure 8:**
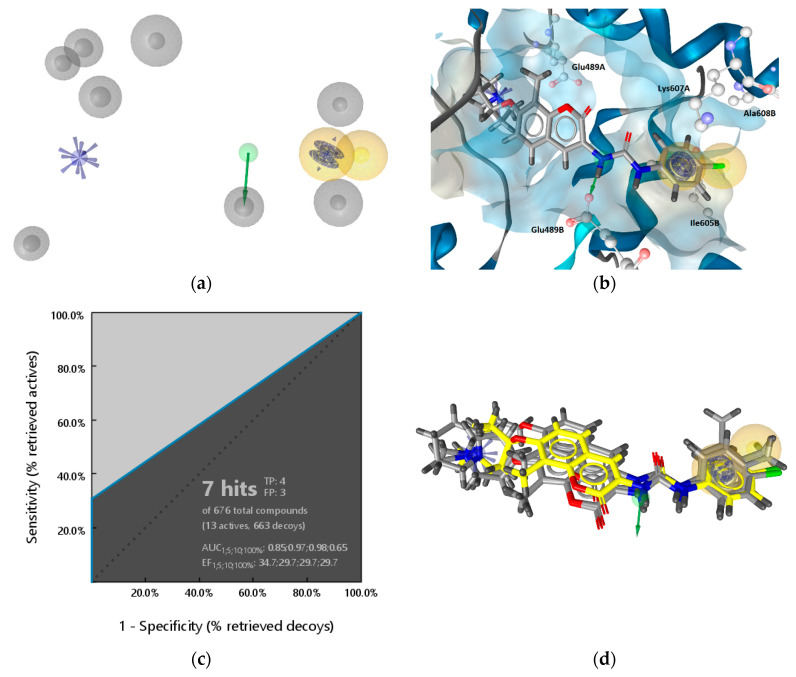
(**a**) The molecular dynamics (MD)-derived structure-based (SB) pharmacophore model prioritized for virtual screening and (**b**) binding mode of **2** with pharmacophore features derived from MD simulations. For clarity, only amino acids interacting with the ligand are shown. The pharmacophore features are as follows: hydrophobic (yellow spheres), aromatic (blue disc), hydrogen bond donor (green arrow), positive ionizable (blue star), exclusion volumes (grey spheres). (**c**) Resulting ROC plot (curve shown in blue), from virtually screening 676 compounds (13 Hsp90 CTD actives and 663 generated decoys) with the SB pharmacophore model. TP = true positives; FP = false positives; AUC = area under the curve; EF = enrichment factor. (**d**) Alignment of four hits with SB-model derived from Hsp90β allosteric CTD domain MD simulations. Compound **2** is highlighted in yellow.

Interestingly, the second most frequently appearing model (176 times) exhibited 5 interaction features, including a directly observed positive ionizable, associated with the tertiary amine and Glu489A, hydrophobic interactions with Ile605B and Ala608A, a H-bond between Glu489B and a urea NH and a cation-π interaction between the chlorophenyl aromatic and Lys607A (Figure 8b). Although Lys607A had been implicated in H-bond interactions in previous studies, this is the first reported observation of the cation-π interaction of Hsp90 CTD inhibitor with Lys607A. This is interesting, since all of the 13 active derivatives contain aromatic substituents in a similar position, including compound **S12** (Supplementary Table S1) that did not match a hydrophobic feature in the LB model, due to the presence of the triazole, which could instead match an aromatic feature in that position.

MD simulations of both novobiocin and compound **2** revealed additional interactions with the predicted allosteric Hsp90 CTD binding site that were not observed in docking studies. Overall, the MD based analyses gave a dynamic view of the potential interactions that could be formed in the Hsp90 CTD allosteric pocket and could prove useful for rationalizing a molecular basis for activity and furthermore supporting the design of compounds using binding sites from MD where those interactions occurred.

### 2.5. Structure-Based Pharmacophore Models Derived from MD

An ensemble of SB pharmacophore models was generated from each frame of the MD trajectory of the Hsp90β CTD with compound **2** using LigandScout 4.3. The common hits approach (CHA) filter [30] was used to obtain a set of the ten most frequently occurring interaction models during the MD simulation. Each SB model was assessed by screening the active Hsp90 CTD inhibitor and decoy libraries. The prioritized SB model selected for the VS campaign is shown in Figure 8a,b, and the ROC curve summarizing the assessment in Figure 8c. The model was the second most frequently occurring and described in detail in Section 2.4. Eight EVs were added automatically to the model based on the location of the interaction partners from the binding site meaning locations on the protein side, where amino acids which formed interactions with the ligand were restricted during VS. In the assessment, the prioritized SB model retrieved four of the 13 active compounds, including compounds **2** and **S1**–**S3** (Supplementary Table S1). The retrieved actives are close analogues of **2** and are shown aligned to the SB model in Figure 8d. Coumarin derivatives with the biphenyl moiety (Supplementary Table S1, compounds **S5**–**S7**) could not match the hydrophobic feature on the right side due to the *meta*-substituted biphenyl moiety, while compounds **S8**–**S12** (Supplementary Table S1) did not match the H-bond donor feature and/or positive ionizable feature. The H-bond donor vector and the aromatic features when considered together, as defined by the model, are highly restrictive, due to defined directionality, and significantly contributed to the selectivity of the model. Disabling the aromatic feature resulted in the retrieval of four more of the actives, **S4**, **S5**, **S7** and **S8**. Furthermore, disabling the EVs and increasing the feature tolerance of the aromatic resulted in the retrieval of 10 of the 13 molecules indicating that all of the actives except **S6**, **S10** and **S12** could match all of the interaction features in the SB model derived from the MD simulation. However, loosening the selectivity of SB model for VS purposes is undesirable, as the hit rate will increase, as well as false positive rates. Similar to the LB model (Figure 2b), the SB model exhibited a desirable low overall hit rate at 1% and 0.5% of the decoy molecules, though it covered less than 50% of the desired active space. This is reflected in the lower EFs (34.7 and 29.7) and AUC values and a curve (blue line), that is closer to the random line compared to the LB model. Furthermore, the SB-model did not retrieve any of the Hsp90 N-terminal domain inhibitors derived from X-ray data.

### 2.6. Comparison of LB and MD-Derived SB Models

The LB and SB models were developed starting from different methods. In the case of LB modeling, bioactive conformations of the ligands were not known. The pharmacophore features were added to the model based on multiple pharmacophore feature alignment experiments, using 200 generated conformations for each molecule. In addition, the selected molecules were synthetically optimized derivatives of novobiocin with established SAR in terms of chemical substituents involved in the desired activity. The resulting LB model could identify all of the common chemical features and the 3D-geometry of those features. In addition, an EV coat was automatically added to the model based on the shape of the aligned molecules.

**Figure 9:**
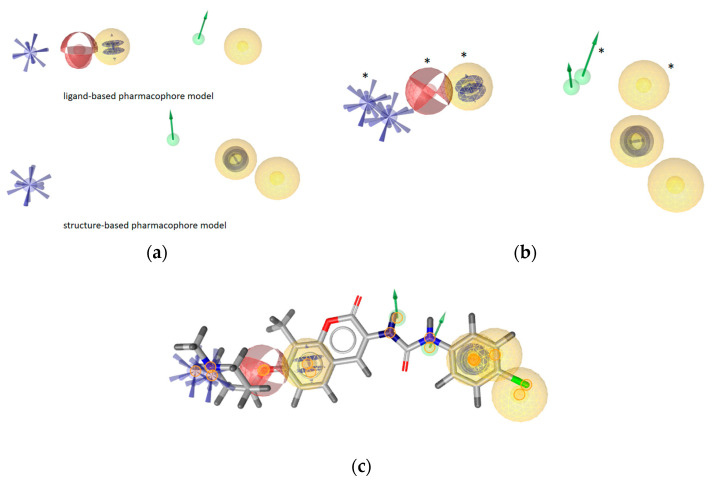
Comparison of the ligand and molecular dynamics(MD)-derived structure-based pharmacophore models prioritized for virtual screening to identify Hsp90 CTD inhibitors. (**a**) Ligand-based (LB) model (above) and structure-based (SB) model below. Exclusion volumes not displayed. (**b**) The LB and MD-SB derived models aligned. Features marked by * belong to the LB model. (**c**) Alignments of both models and compound **2**. The pharmacophore features are as follows: hydrophobic (yellow spheres), aromatic (blue disc), hydrogen bond donor (green arrow), positive ionizable (blue star).

In contrast to the LB model, the features in the SB model rely on interaction partners from the binding site. An interaction feature is added to the model only when there is a complementary partner in the binding site in the correct geometry between the ligand and protein. EVs are added to the model based on the position of the binding partners in the active site. Based on the different approaches, the LB and SB models are expected to have different interaction features and EVs (Figure 9). At the same time, since compound **2** was involved in the generation of both models, one expects to find similar features in both models. On the other hand, it is not clear that the Hsp90 inhibition of these inhibitors is a result of direct binding at an allosteric CTD site, since so far there are no X-ray or cryo-EM resolved complexes of Hsp90 CTD binding sites and no assays for direct measurement. Therefore, 3D-interaction features, which are similar in the two models, offer a new way to rationalize a molecular basis for CTD activity. Using the LB and SB models to identify new active compounds provides a way to make sense of observed interactions and use them for identifying new tool compounds to probe the mechanism of action based on rational design approaches.

Figure 9 displays the LB and MD-derived SB models prioritized for VS (Figure 9a), the models aligned (Figure 9b) and compound **2** aligned to both models (Figure 9c). Common features, though not perfectly aligned 3D-geometries, are positive ionizable, H-bond donors and hydrophobic interactions. However, a closer inspection of the aligned models shows that positions of the hydrophobics are unique with varying proximities to the H-bond donor. In addition, both models contained restrictive directional aromatics (LigandScout aromatic features can capture parallel and orthogonal interactions) though in distinct geometries. In the LB model, the aromatic feature is in closer proximity to the positive ionizable (6.45 Å) than in the SB-model, where it is positioned near the H-bond donor. The optimal distance and angle between the *N*-methylpiperidine and the biaryl side chain have been studied in a library of novobiocin core analogs [31]. The resulting LB model based on some of those optimized structures reflects both features. However, the aromatic/hydrophobic and H-bond acceptor in the LB model are not present in the SB model, because there were no binding partners in optimal distances and geometries in that binding site frame of the MD trajectory. However, the other aromatic feature appears in the SB-model due to the cation-π interaction with Lys607A as a binding partner.

Compound **2** can match the features of both models, as displayed in Figure 3a and Figure 8d. However, the H-bond donor is matched by different urea NHs (Figure 9c). In addition, though the aromatic substituents in compound **2** can match aromatic features in both models, they cannot be matched at the same time, due to the directionality restriction of the aromatic (Figure 9c).

Based on these comparisons, the LB and SB models are expected to retrieve some of the same and some different molecules during a VS campaign. In addition, the models are not only useful as queries for VS, but offer an advantage because they contain information that is easily tractable to the molecular structure, which is important for rationalizing molecular design and optimization- chemistry decision support.

### 2.7. Virtual Screening

Although most of the known Hsp90 CTD inhibitors are novobiocin analogues [13], some were also discovered by virtual screening, employing ligand-based pharmacophore models [25], or 3D similarity searching [32]. Our unique approach involved a MD-derived SB-pharmacophore derived from a predicted apo allosteric Hsp90 CTD (Figure 8) and an LB model (Figure 2), as queries to virtually screen a library of 556,000 diverse commercially available compounds. The goal was to identify compounds with unique scaffolds, relative to novobiocin derivatives, that exhibit antiproliferative activity in cancer cell lines and act as Hsp90 CTD inhibitors. Compounds retrieved as hits were required to match all features of the models including EVs. The LB and SB models retrieved 102 and 324 virtual hits, respectively, revealing overall hit rates under 1%, and as expected, some hits were identified by both models. The LB model retrieved fewer hits than the SB model due to the larger number of pharmacophore features and a heavier EV coat.

Eight compounds were prioritized for purchase and experimental assessment based on pharmacophore fit scores, scaffolds, availability, and budget. Coumarin and biphenyl substructures were excluded from the prioritized hits. Table 1 summarizes the structures of the purchased virtual hits **5**–**12** along with their relative pharmacophore fit scores. Higher pharmacophore fit scores represent a better fit to a model. Compounds **8**, **9**, and **11** were retrieved by both models, whereas compounds **5**–**7**, **10**, and **12** were retrieved only by the LB model. Compounds **6** and **7** had the lowest pharmacophore scores among the eight prioritized hits (Table 1). Analysis of the virtual hit scaffolds revealed that all contained primary or tertiary amines and (hetero)aromatic moieties, with different linkers connecting these two essential features (Table 1). 

### 2.8. Biological Evaluation

**Figure 10:**
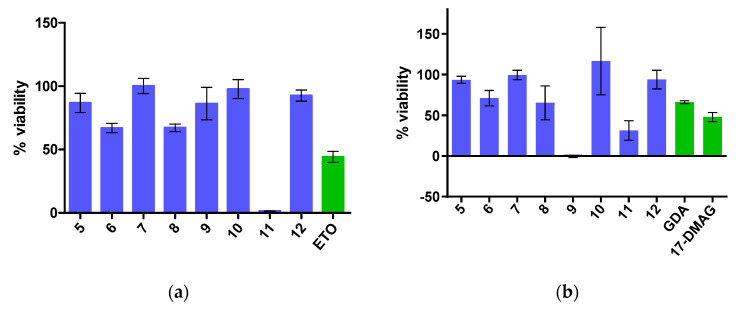
Cell viability of (**a**) Hep G2 and (**b**) MCF-7 cells in MTS assay after treatment with compounds **5**–**12**, and etoposide (ETO) at 50 µM concentration or geldanamycin (GDA) at 10 nM concentration and 17-DMAG at 1 µM concentration. Data are means ± SD of three independent experiments performed in triplicate.

Eight compounds, which were identified as potential Hsp90 CTD inhibitors by virtual screening using LB and MD-SB pharmacophore models, were tested for their effect on cell viability using an MTS assay in Hep G2 liver cancer and MCF-7 breast cancer cell lines, with high overexpression of Hsp90 (Figure 10). Compound **11** displayed IC_50_ values of 27.9 ± 0.7 μM and 44.8 ± 3.6 μM against the Hep G2 and MCF-7 cell lines, respectively (Supplementary Figures S1 and S2). Moreover, compound **9** was found to be more potent than **11** against the MCF-7 cell line (IC_50_ value of 31.8 ± 3.0 μM) (Supplementary Figure S3). Compounds **6** and **8** showed weak antiproliferative activity, while compounds **5**, **7**, **10** and **12** were devoid of antiproliferative activity at 50 µM (Figure 10, Supplementary Table S2). Compounds **9** and **11** were far more potent in the antiproliferation assays than novobiocin, the starting point for the novobiocin Hsp90 CTD inhibitor analogues. However, further structural optimization of early virtual screening hits **9** and **11** is needed to reach the level of activity of compound **2**. In addition to the improvement of potency, physico-chemical and ADME properties of compounds will be evaluated and optimized, and potential toxicity issues, such as unwanted hERG inhibition, will be monitored.

**Figure 11:**
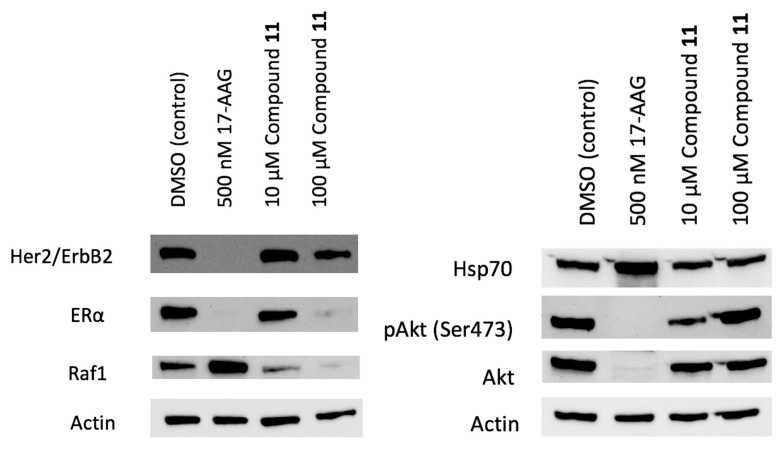
Representative Western blot analyses 24 h after treatment with **11** at concentrations of 10 and 100 μM and 17-AAG at concentration 500 nM in MCF-7 cells vs. DMSO (vehicle, negative control). Marked and dose-dependent decreases of Hsp90 clients were observed without the significant induction of Hsp70 levels.

All eight virtual hits were tested for their ability to inhibit the Hsp90-dependent refolding of denatured luciferase in a PC3 MM2 cell line at 50 μM. Only compound **11** showed significant activity (Supplementary Figure S4), confirming that its antiproliferative effect observed in Hep G2 and MCF-7 cell lines was due to Hsp90 inhibition. The IC_50_ value against PC3 MM2 cells determined in a dose-response experiment was 97.1 ± 8.8 μM (Supplementary Figure S5). Therefore, compound **11** was further investigated for its ability to induce the degradation of Hsp90-dependent clients in the MCF-7 cell line. As presented in the Western blot (Figure 11), compound **11** induced the degradation of Hsp90 substrates HER2, ERα and Raf1, while a weaker effect was observed in the case of Akt. The degradation of pAkt was dose-dependent, but surprisingly, it was higher at a lower concentration of 10 µM. Importantly, the level of Hsp70 remained unchanged at concentrations 10 and 100 μM. These results highlight compound **11** as Hsp90 inhibitor that induces the degradation of client proteins without induction of the heat shock response, which is one of the hallmarks of Hsp90 CTD inhibitors.

### 2.9. Binding Mode of Compound **11** in the Putative Allosteric Hsp90 CTD Binding Site

**Figure 12:**
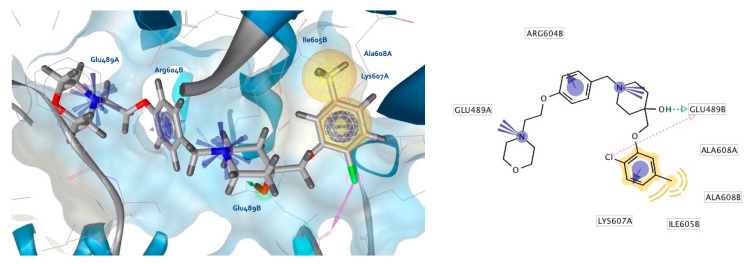
Interactions of compound **11** with the Hsp90 CTD binding site from the MD simulation used to derive the SB-model for virtual screening. The pharmacophore features are as follows: hydrophobics (yellow spheres), aromatics (blue discs), hydrogen bond donor (green arrow), positive ionizables (blue stars), halogen bond (pink arrow).

**Figure 13:**
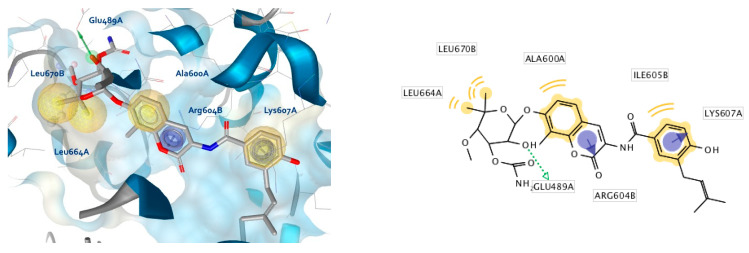
Interactions of novobiocin with the Hsp90 CTD binding site from the MD simulation used to derive the SB-model for virtual screening. The pharmacophore features are as follows: hydrophobics (yellow spheres), aromatics (blue discs), hydrogen bond donor (green arrow).

**Figure 14:**
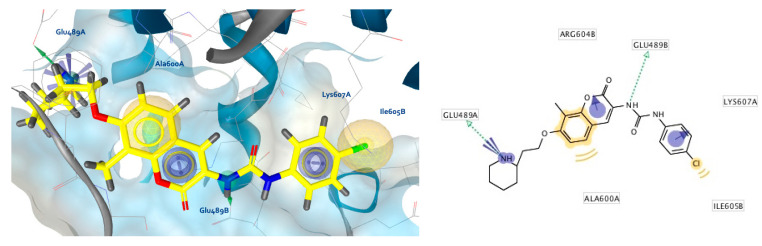
Interactions of compound **S3** (Supplementary Table S1), an active novobiocin derivative, with the Hsp90 CTD binding site from the MD simulation used to derive the SB-model for virtual screening. The pharmacophore features are as follows: hydrophobics (yellow spheres), aromatics (blue discs), hydrogen bond donor (green arrow), positive ionizable (blue stars).

Compound **11** was retrieved by both the LB and SB pharmacophore models in the study, similar to test screens resulting in compounds **2**, and **S1**–**S3** from the actives library. Figure 12 shows compound **11** in the MD simulation frame from which the SB pharmacophore model for virtual screening was derived. In addition, novobiocin (Figure 13) and the highly active CTD inhibitor **S3** (Figure 14) from the actives dataset were also injected into the same site, to explore interactions and compare them to compounds **2** and **11**. The morpholine nitrogen of compound **11** is within proximity of Glu489A and can form a strong ionic interaction, while the hydroxyl group on the piperidine ring forms H-bond with Glu489B, and the Lys607A side chain is engaged in a cation-π interaction with the chlorophenyl ring. Moreover, hydrophobic interactions between compound **11** and Ile605B, Ala608A, and Ala608B were formed. In contrast to the interactions of compound **2** (Figure 8b), a second positive ionizable between the piperidine nitrogen and Glu489B, a potential halogen bond between the chlorine atom and Glu489B backbone carbonyl, and an additional cation-π interaction with Arg604B was observed. Additional MD simulation of compound **11** in the putative Hsp90 CTD binding site is needed to study the most frequently occurring binding mode that will be used for further optimization studies.

Novobiocin and the active analogue **S3** (Supplementary Table S1) formed similar interactions as compound **11**, including Glu489A (hydrogen bond or positive ionizable) and cation-π interactions with Arg604B and Lys607A (Figure 13 and Figure 14). Although a different binding site was previously reported for novobiocin and its analogues [24], novobiocin, and active novobiocin analogues **2** and **S3** could form similar interactions in the same binding site as compound **11**, that was identified using a SB model derived from the MD trajectory.

Though these studies do not confirm the mode of action of these inhibitors at the Hsp90 CTD domain, the identification of compound **11** based on interactions derived from the predicted binding site provide a useful basis and rationale for the use of these binding sites for the structure-based optimization of analogues with improved Hsp90 CTD inhibition, as well as the use of SB pharmacophore models derived from MD simulations of active Hsp90 CTD inhibitors for virtual screening campaigns to identify new tool compounds.

## 3. Materials and Methods

### 3.1. Software

Allosteric pocket prediction, MD-trajectory analysis, VS, generation and assessment of the SB- and LB-pharmacophore models and calculation of virtual screening libraries were performed using LigandScout 4.3 Expert, available from Inte:Ligand GmbH [29,33]. Multi-conformational compound libraries for virtual screening were created with LigandScout algorithms, i:Con (conformation generation) [34,35] and idbgen (virtual screening library generation). VS and common hits approach filtering was performed using iScreen and the CHA nodes in the Inte:Ligand Expert KNIME Extensions [36], as integrated for use in the KNIME Analytics Platform [37]. NAMD (version 2.9) [38] and CHARMM22 [39,40] were used for MD simulations. The structure of each energy minimized complex for MD simulations was prepared using psfgen in VMD (version 1.9.1.) [41]. AutoDock Vina 1.1 [42] was used for molecular docking as built-in in LigandScout 4.3 Expert.

### 3.2. Virtual Compound Library Preparation

Three virtual compound libraries were prepared for VS: (1) a selected set of experimentally measured Hsp90 CTD actives, (2) a calculated set of decoy molecules and (3) a merged set of compounds available from commercial providers for hit identification via VS with prioritized pharmacophore models. For the Hsp90 CTD actives library, structures of 13 novobiocin analogues Hsp90 CTD inhibitors with submicromolar activity were manually selected from scientific publications (Supplementary Table S1) [22,23,43]. A second library of 663 decoy molecules was generated based on each of the 13 Hsp90 CTD inhibitors, by submitting their structures to the DUDE decoy online generator [44], which resulted in 51 decoy molecules (SDF format) per compound with similar 1D physicochemical properties, but dissimilar 2D topology in comparison to the 13 active compounds. Decoys are hypothetical structures that are unlikely to display activity at the target protein, but have not been tested experimentally. They are useful for comparing 3D-pharmacophore models using a receiver operating (ROC) curve analysis when not enough suitable negative or inactive compounds experimentally evaluated are available.

A third library of 556,000 compounds from commercial providers was prepared based on the diversity sets from Enamine, Asinex, ChemBridge, Maybridge, LifeChemicals, Vitas-M and KeyOrganics. Libraries were downloaded in SDF format, merged and duplicates removed using the LigandScout Database Merger and Duplicates Remover nodes, as implemented in the Inte:Ligand Expert KNIME Extensions [36].

For each of the three libraries, a maximum of 200 conformations were generated for each molecule using LigandScout’s iCon algorithm with the default “BEST” settings (max. number of conformers per molecules: 200, timeout (s): 600, RMS threshold: 0.8, energy window: 20.0, max. pool size: 4000, max. fragment build time: 30). Each library was saved in LDB (LigandScout database format) using LigandScout’s idbgen algorithm with default settings (write all properties and remove duplicates).

### 3.3. Ligand-Based Pharmacophore Modeling

Thirteen Hsp90 CTD inhibitors, including compound **2** (Figure 1), displaying IC_50_ values between 0.13 and 0.5 μM against the SKBr-3 breast cancer cell line (Supplementary Table S1) were used for creation of ten LB pharmacophore models in LigandScout 4.3. A maximum of 200 conformations was generated for each compound, as described in the library generation section. The models were generated using the following ligand-based pharmacophore creation settings: scoring function: pharmacophore fit and atom overlap; pharmacophore type: merged feature pharmacophore; number of omitted features for merged pharmacophore: 4; partially matching features optional, threshold (%): 10.0; feature tolerance scale factor: 1.0; maximum number of result pharmacophores: 10. Create exclusion volumes and apply custom feature definitions was ticked on in the tick box. Creation of an exclusion volumes coat around the alignment of the ligands was also enabled for each of the models. All ligands of the training set were automatically aligned to the generated pharmacophore models. The resulting LB pharmacophore models were tested for their performance in distinguishing the active and decoy molecules. The best performing model was selected for VS of the commercially available compounds.

### 3.4. Hsp90 CTD Allosteric Binding Pocket Prediction

A cryoEM structure of full-length human Hsp90β (PDB Code: 5FWK) [28] was downloaded from the RCSB Protein Data Bank [45]. Co-chaperone Cdc37 and client protein CDK4 were deleted from the complex, while ATP and Mg^2+^ ions were retained. LigandScout’s Calculate Pockets algorithm (default settings) was used for the detection of the allosteric binding site at the interface of two Hsp90 CTDs. LigandScout places isosurfaces on detected binding sites. Recommended binding sites based on size and buriedness are colored orange. Several binding sites were identified in the CTD domain, while only a large binding site was recommended. The site was selected and a binding site was created. LigandScout places a yellow box around the binding site, enabling the user click on the box, zoom into the pocket and use it for additional modeling experiments.

### 3.5. Molecular Docking

Novobiocin and a library of 13 Hsp90 CTD inhibitors, described above, were docked to the identified allosteric binding site at the Hsp90 CTD using AutoDock Vina 1.1 [42] as built-in in LigandScout 4.3. The following default settings were used for docking: exhaustiveness: 8, max. number of modes: 9, max. energy difference: 3. Poses for novobiocin and compound **2** were prioritized for starting structures for all-atom MD simulations based on AutoDock’s binding affinity score and LigandScout’s total number of pharmacophore feature interactions calculation. Compound **2** was selected for further studies due to its potency and lower molecular weight compared to other Hsp90 CTD inhibitors in the library.

### 3.6. Molecular Dynamics Simulations

The MD package NAMD (version 2.9) [38] and CHARMM22 force field [39,40] were used for MD simulations using the cryoEM structure of full-length human Hsp90β (PDB Code: 5FWK) [28]. Molecular mechanics parameters for novobiocin and compound **2** were estimated using ParamChem tool [46,47,48]. Steepest descent (10,000 steps) and adopted basis Newton−Raphson (10,000 steps) energy minimizations were first performed to remove atomic clashes and optimize the atomic coordinates of the Hsp90β−novobiocin and Hsp90β−**2** complexes. The structure of the energy minimized complex for MD simulation was prepared using psfgen in VMD (version 1.9.1.) [41]. The protein-ligand complex was embedded in box of TIP3P water molecules and the system neutralized by addition of NaCl. The NPT ensemble employing periodic boundary conditions was used for MD simulations. Temperature (300 K) and pressure (1 atm) were controlled by the use of Langevin dynamics and Langevin piston methods, respectively. Short- and long-range forces were calculated every 1 and 2 time steps, respectively, with a time step of 2.0 ps. Electrostatic interactions were calculated with the smooth particle mesh Ewald method [49]. A cut off of 12 Å was set for the short-range interactions. The SHAKE algorithm was used to keep all chemical bonds between hydrogen and heavy atoms fixed [50]. The MD simulations consisted of (i) solvent equilibration for 100 ps with protein and ligand constrained harmonically around the initial structure, (ii) equilibration of the complete system for 500 ps with protein and ligand released, and (iii) an unconstrained 20 ns production run that was later used for structure-based pharmacophore modeling.

### 3.7. Structure-Based Pharmacophore Modeling

The MD trajectory of the full length Hsp90β dimer in complex with compound **2** was used for chemical feature interaction analysis using LigandScout 4.3 Expert. The first frame from the MD trajectory was saved in PDB format. The first frame of the trajectory (PDB format) and the MD trajectory files (CHARMM format) are both needed as input for the creation of an ensemble of structure-based pharmacophore models. LigandScout can extract chemical feature interactions between proteins and ligands in a click of a button [29]. LigandScout 4.3 Expert can generate SB interaction models for each frame of a MD trajectory. The Inte:Ligand Expert KNIME MD trajectory node was used to calculate 2000 structure-based (SB) pharmacophore models from the 20 ns MD simulation. The common hits approach (CHA) [30] filter as implemented in the Inte:Ligand Expert KNIME extensions MD analysis tools was used to identify 10 of the most frequently occurring and unique pharmacophore models. All ten models were evaluated by virtually screening the libraries of active and decoy molecules. The best performing SB model was selected for VS, the library of commercially available compounds.

### 3.8. Virtual Screening

The selected LB and SB pharmacophore models were used to query the library of 556,000 commercially available diverse compounds, prepared as described using LigandScout 4.3 Expert. The settings included: Scoring Function: Pharmacophore-Fit, Screening Mode: Match all query features; Retrieval mode: Stop after first matching conformation; Max. number of omitted features: 0 and Check exclusion volumes: true. Virtual hits were ranked according to Relative Pharmacophore fit score. Higher ranking hits have a fit score closer to 1.0. The hits were visually inspected and the highest ranked compounds based on fit scores were selected and purchased from commercial vendors.

### 3.9. MTS Assay

Antiproliferative activities of compounds in MCF-7 and Hep G2 cell lines were determined with MTS (Promega, Madison, WI, USA) assay according to manufacturer instructions. Briefly, MCF-7 and Hep G2 cells were cultured in Eagle’s MEM medium (Gibco, Thermo Fisher Scientific, Waltham, MA, USA), supplemented with 10% fetal bovine serum (Gibco, Thermo Fisher Scientific, Waltham, MA, USA), 100 U/mL penicillin (Sigma-Aldrich, St. Louis, MO, USA), 100 µg/mL streptomycin (Sigma-Aldrich, St. Louis, MO, USA), and 2 mM L-glutamine (Sigma-Aldrich, St. Louis, MO, USA). The cells were incubated at 37 °C in a 5% CO_2_ atmosphere. The cells were plated in 96-well plates at a density of 2000 cells per well, and incubated for 24 h. Then, the cells were treated with selected compounds, positive control (10 nM geldanamycin and 1 µM 17-DMAG or 50 µM etoposide) or vehicle control (0.5% DMSO) and incubated for 72 h. CellTiter96^®^ Aqueous One Solution Reagent (10 µL; Promega, Madison, WI, USA) was then added to each well, and the plate was incubated for another 3 h. Absorbance was measured with a BioTek’s Synergy™ 4 Hybrid Microplate Reader (Winooski, VT, USA). Independent experiments were repeated three times, each performed in triplicate. Statistical significance (*p* < 0.05) was calculated with two-tailed Welch’s *t*-test between treated groups and DMSO. IC_50_ values (concentration of the compound that gives a half-maximal response) are given as average values from the independent measurements, and were determined using GraphPad Prism 5.0 software (San Diego, CA, USA).

### 3.10. Luciferase Refolding Assay

Hsp90-dependent luciferase refolding assay was performed in PC3 MM2 cells expressing firefly luciferase [51]. Briefly, cells were cultured in Dulbecco’s modified Eagle’s medium (Gibco, Thermo Fisher Scientific, Waltham, MA, USA), supplemented with 5 µg/mL puromycin (InvivoGen, San Diego, CA, USA), 100 U/mL penicillin (Sigma-Aldrich, St. Louis, MO, USA), and 100 µg/mL streptomycin (Sigma-Aldrich, St. Louis, MO, USA), and 10% fetal bovine serum (Gibco, Thermo Fisher Scientific, Waltham, MA, USA), at 37 °C and under 5% CO_2_. Cell pellets were collected from 80–90% confluent flasks and suspended in pre-warmed media (50 °C) for 2 min. The cells were plated in 96-well plates at a density of 50,000 cells per well in the presence of selected compounds, vehicle control (1% DMSO) or positive control (50 µM geldanamycin and Hsp90 CTD inhibitor). The plates were then incubated for 60 min at 37 °C to allow for luciferase refolding. Moreover, 100 µL of ONE-Glo™ Luciferase Assay System (Promega, Madison, WI, USA) was then added to each well of the plate and incubated for another 5 min. Luciferase activity was determined by measuring luminescence with BioTek’s Synergy™ 4 Hybrid Microplate Reader (Winooski, VT, USA). Independent experiments were repeated three times, each performed in triplicate. Statistical significance (*p* < 0.05) was calculated with two-tailed Welch’s *t*-test between treated groups and DMSO. IC_50_ values (concentration of the inhibitor that gives a half-maximal response) are given as average values from the independent measurements, and were determined using GraphPad Prism 5.0 software (San Diego, CA, USA).

### 3.11. Western Blot for MCF-7 Cells

MCF-7 cells were cultured in Dulbecco’s modified Eagle’s MEM medium (Gibco, Thermo Fisher Scientific, Waltham, MA, USA), supplemented with 100 U/mL penicillin (Sigma-Aldrich, St. Louis, MO, USA), and 100 µg/mL streptomycin (Sigma-Aldrich, St. Louis, MO, USA), and 10% FBS (Gibco, Thermo Fisher Scientific, Waltham, MA, USA) at 37 °C and under 5% CO_2_. Cells were treated with 10 µM compound **11**, 100 µM compound **11**, 500 nM 17-AAG or DMSO dissolved in this medium for 24 h. After 24 h of drug exposure, cells were washed with 1 × PBS (137 mM NaCl, 2.7 mM KCl, 10 mM Na_2_PO_4_, 1.8 mM KH_2_PH_4_, pH 7.4), and then lysed in M-PERTM Mammalian Protein Extraction Reagent (Thermo Fisher Scientific, Waltham, MA, USA), containing 1:100 Phosphatase Inhibitor Cocktail 2, 1:100 Phosphatase Inhibitor Cocktail 3 (Sigma-Aldrich, St. Louis, MO, USA), 1:100 Protease Inhibitor Cocktail Set III, EDTA-Free (Calbiochem, MilliporeSigma, Burlington, MA, USA), and 1 mM phenylmethyl sulfonyl fluoride. Cell lysates were centrifuged at 10,000 rcf for 10 min at 4 °C. Supernatants were collected and subject to BCA assay (PierceTM BCA Protein Assay Kit, Pierce, Thermo Fisher Scientific, Waltham, MA, USA) for protein concentration determination. Samples (30 µg/each) were loaded into the wells of a 4–20% Mini-PROTEAN TGXTM Precast Gel (Bio-Rad, Hercules, CA, USA), electrophoresed at 200 V, and transferred to an Immun-Blot PVDF Membrane using a Trans-Blot Turbo^TM^ Transfer System (Bio-Rad, Hercules, CA, USA). Membranes were blocked in 7% non-fat milk for 1 h at rt, prior to exposure to primary antibody solutions. Primary antibodies used in these experiments included HER2/ErbB2 (29D8) Rabbit mAb (1:1000, Cell Signaling, Danvers, MA, USA), Estrogen Receptor α (D8H8) Rabbit mAb (1:1000, Cell Signaling), β-Actin (13E5) Rabbit mAb (1:1000, Cell Signaling), Raf-1 (C-12) sc-133 Rabbit polyclonal IgG (1:1000 Santa Cruz Biotechnology, Dallas, TX, USA), HSP70/HSP72 (C92F3A-5) (1:1000, Enzo Life Sciences, Farmingdale, NY, USA), Phospho-Akt (Ser473) (D9E) XP^®^ Rabbit mAb (1:1000, Cell Signaling), and Akt Rabbit Ab (9272S) (1:1000, Cell Signaling). Each solution of primary antibody contained 0.3% sodium azide. Secondary antibodies used in these experiments were Anti-rabbit IgG, HRP-linked antibody (1:2000, Cell Signaling) and Goat Anti-mouse Ig, Human ads-HRP (1:2000, SouthernBiotech, Birmingham, AL, USA). Blots were visualized using a ChemiDoc Imaging System (Bio-Rad, Hercules, CA, USA).

## 4. Conclusions

In conclusion, ligand and molecular dynamics-derived structure-based pharmacophore models were developed to identify a new structural class of Hsp90 CTD inhibitors by virtual screening. The ligand-based models were based on known potent novobiocin derivatives. The structure-based model was uniquely derived from MD simulations of a potent inhibitor in a predicted allosteric apo binding pocket in the CTD domain of Hsp90β. Compounds **9** and **11** were identified by both pharmacophore models and displayed significant antiproliferative activity in breast and liver cancer cell lines. Furthermore, compound **11** inhibited the Hsp90-dependent refolding of denatured luciferase, and was shown to induce the degradation of Hsp90 substrates without the induction of the heat shock response, an important characteristic of Hsp90 CTD inhibitors.

The 3D-pharmacophore models that retrieved compound **11** and the associated binding sites from the MD trajectory can be used successfully for further virtual screening campaigns and the de novo design of novel Hsp90 CTD inhibitors and compounds with antiproliferative activity in cancer cell lines. Moreover, compound **11**, with a unique scaffold, was successfully identified as a promising starting point for further optimization towards the evaluation and development of tool compounds to probe the mode of action of Hsp90 CTD inhibition and develop candidates for anticancer drugs.

Generally, structure-based approaches using apo structures of allosteric binding sites have been met with varying success. This study highlights the potential of high-resolution cryoEM apo structures, in combination with molecular dynamics simulations and 3D-chemical feature-based pharmacophore modeling for difficult targets, such as the highly dynamic Hsp90 chaperone. Structure-based optimization of compound **11** towards more potent analogues is underway and will be reported in the near future.

## Figures and Tables

**Table 1:** Structures of virtual screening hits identified by ligand- and/or structure-based pharmacophore models. Higher pharmacophore fit scores indicate a closer fit to the model. Compounds not retrieved by the SB model are designated with /.

Compound	Structure	LB Pharmacophore Fit Score	SB Pharmacophore Fit Score
**5**	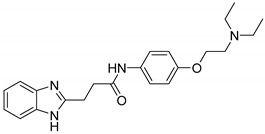	0.93	/
**6**	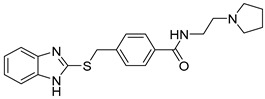	0.90	/
**7**	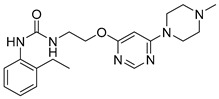	0.92	/
**8**	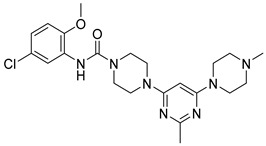	0.91	0.94
**9**	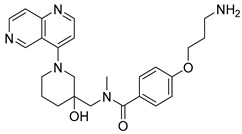	0.93	0.93
**10**	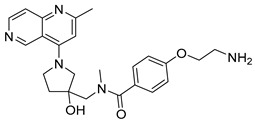	0.94	/
**11**	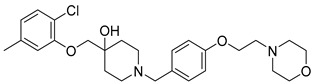	0.93	0.94
**12**	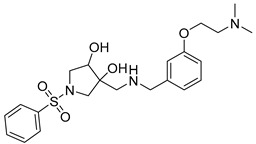	0.94	/

## Supplementary Materials

Supplementary materials can be found at https://www.mdpi.com/1422-0067/21/18/6898/s1.

## Abbreviations

SB	Structure-Based
LB	Ligand-Based
Hsp	Heat Shock Protein
MD	Molecular Dynamics
CTD	C-Terminal Domain
NTD	N-Terminal Domain
VS	Virtual Screening
