# Supplementary material for: Discovery of Novel Hsp90 C-Terminal Inhibitors Using 3D-Pharmacophores Derived from Molecular Dynamics Simulations
Source: Int J Mol Sci. 2020 Sep 20;21(18):6898. doi: 10.3390/ijms21186898 (PMC7555175; doi:10.3390/ijms21186898)
Supplement: Supplementary file 1 [file ijms-21-06898-s001.pdf]

Supplementary Materials

Discovery of Novel Hsp90 C-Terminal Inhibitors Using 3D-Pharmacophores Derived from Molecular Dynamics Simulations

Tihomir Tomašič [1,*], Martina Durcik [1], Bradley M. Keegan [2], Darja Gramec Skledar [1], Živa Zajec [1], Brian S. J. Blagg [2] and Sharon D. Bryant [3]

[1] University of Ljubljana, Faculty of Pharmacy, Aškerčeva 7,1000 Ljubljana, Slovenia; martina.durcik@ffa.uni-lj.si (M.D.); darja.gramec-skledar@ffa.uni-lj.si (D.G.S.); ziva.zajec@ffa.uni-lj.si (Ž.Z.)

[2] Department of Chemistry and Biochemistry, The University of Notre Dame, 305 McCourtney Hall, Notre Dame, IN 46556, USA; bkeegan@nd.edu (B.M.K.); bblagg@nd.edu (B.S.J.B.)

[3] Inte:Ligand Softwareentwicklungs- und Consulting GmbH, Mariahilferstrasse 74B, 1070 Vienna, Austria; bryant@inteligand.com

* Correspondence: tihomir.tomasic@ffa.uni-lj.si; Tel.: +386-1-4769-556

Table S1. Structures and antiproliferative activities in SKBr3 cell line of Hsp90 CTD inhibitors **2** and **S1–S12** used as a training set for ligand-based (LB) pharmacophore model creation. Each ligand is shown aligned to the resulting LB model (exclusion volumes are not displayed).

Cpd	Structure	SKBr3 IC$_{50}$ (μM)	Alignment with the 3D Ligand-Based Pharmacophore Model
**2**	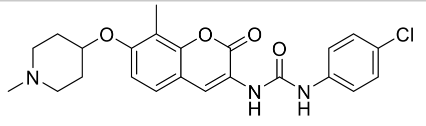 CC1=C2C(C=C(NC(NC3=CC=C(Cl)C=C3)=O)C(O2)=O)=CC=C1OC4CCN(C)CC4	0.17 [1]	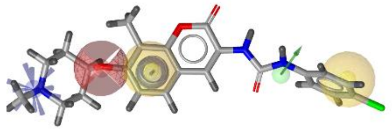
**S1**	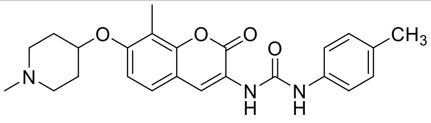 CC1=C2C(C=C(NC(NC3=CC=C(C)C=C3)=O)C(O2)=O)=CC=C1OC4CCN(C)CC4	0.27 [1]	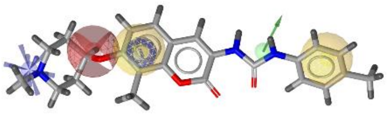
**S2**	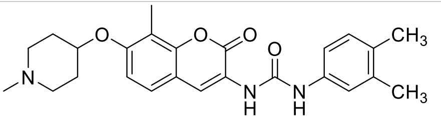 CC1=C2C(C=C(NC(NC3=CC(C)=C(C)C=C3)=O)C(O2)=O)=CC=C1OC4CCN(C)CC4	0.31 [1]	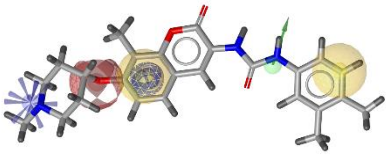
**S3**	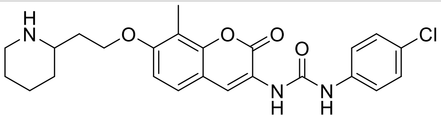 CC1=C2C(C=C(NC(NC3=CC=C(Cl)C=C3)=O)C(O2)=O)=CC=C1OCCC4NCCCC4	0.21 [1]	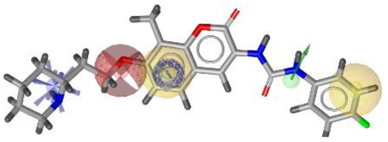
**S4**	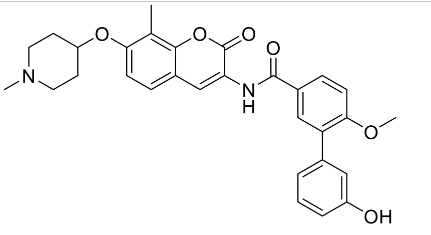 CC1=C2C(C=C(NC(C3=CC(C4=CC=CC(O)=C4)=C(OC)C=C3)=O)C(O2)=O)=CC=C1OC5CCN(C)CC5	0.31 [1]	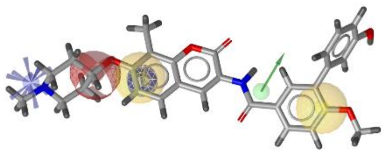

**S5**	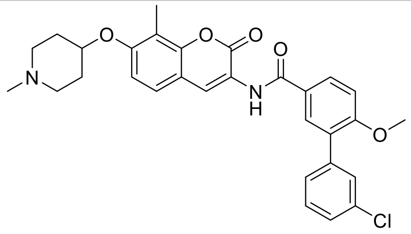<br>CC1=C2C(C=C(NC(C3=CC(C4=CC=CC(Cl)<br>=C4)=C(OC)C=C3)=O)C(O2)=O)=CC=C1OC<br>5CCN(C)CC5	0.13 [1]	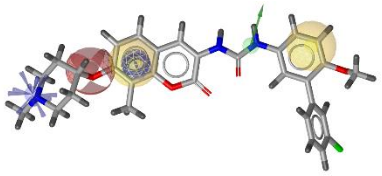
**S6**	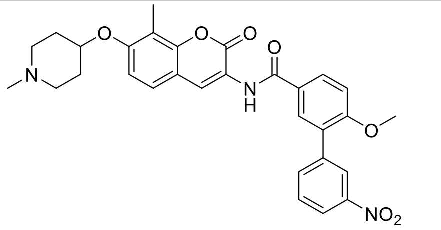<br>CC1=C2C(C=C(NC(C3=CC(C4=CC=CC([N+]([O-<br>])=O)=C4)=C(OC)C=C3)=O)C(O2)=O)=CC=C1OC<br>5CCN(C)CC5	0.16 [1]	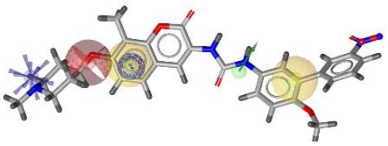
**S7**	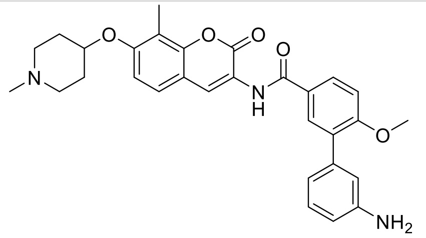<br>CC1=C2C(C=C(NC(C3=CC(C4=CC=CC(N)=C4)<br>=C(OC)C=C3)=O)C(O2)=O)=CC=C1OC5CCN(C<br>)CC5	0.36 [1]	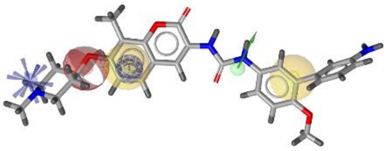
**S8**	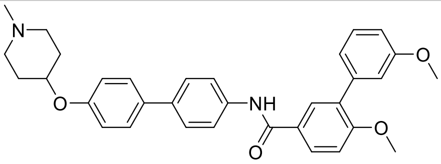<br>CN(CC1)CCC1OC(C=C2)=CC=C2C3=CC=C(NC<br>(C4=CC(C5=CC=CC(OC)=C5)=C(OC)C=C4)=O)<br>C=C3	0.47 [2]	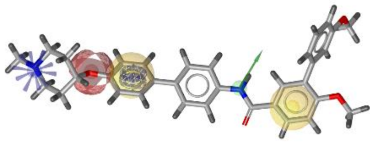
**S9**	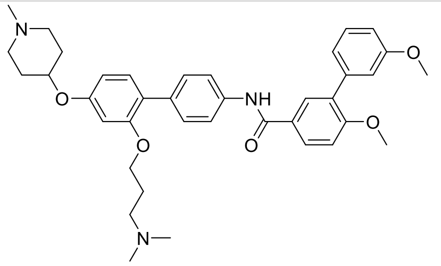<br>CN(CC1)CCC1OC(C=C2)=CC(OCCCN(C)C)=C2C3<br>=CC=C(NC(C4=CC(C5=CC=CC(OC)=C5)=C(OC)C<br>=C4)=O)C=C3	0.39 [2]	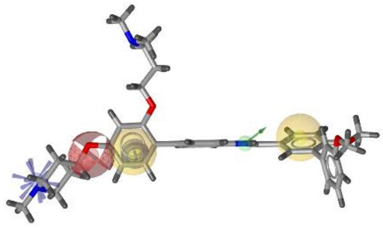
**S10**	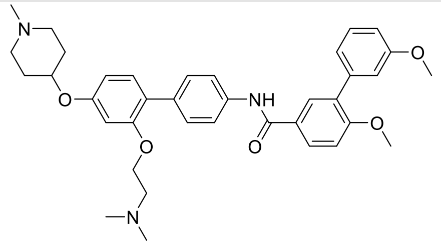<br>CN(CC1)CCC1OC(C=C2)=CC(OCCN(C)C)=C2C3=<br>CC=C(NC(C4=CC(C5=CC=CC(OC)=C5)=C(OC)C=<br>C4)=O)C=C3	0.34 [2]	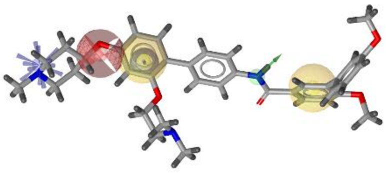

**S11**	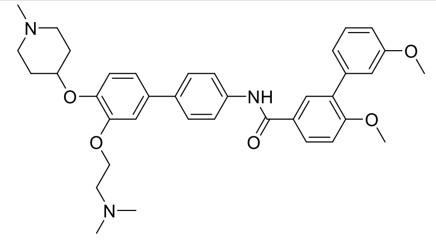 CN(CC1)CCC1OC(C=C2)=C(OCCN(C)C)C=C2C3=CC=C(NC(C4=CC(C5=CC=CC(OC)=C5)=C(OC)C=C4)=O)C=C3	0.40 [2]	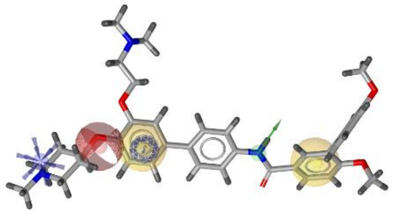
**S12**	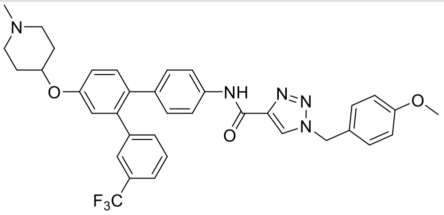 CN(CC1)CCC1OC(C=C2)=CC(C3=CC=CC(C(F)(F)F)=C3)=C2C4=CC=C(NC(C5=CN(CC6=CC=C(OC)C=C6)N=N5)=O)C=C4	0.50 [3]	/

Table S2. Cell viability of Hep G2 and MCF-7 cells in MTS assay after treatment with compounds **5**–**12**. Data are means ± SD of three independent experiments performed in triplicates.

Compound	Hep G2 % Viability	MCF-7 % Viability
**5**	86.8 ± 7.6	93.5 ± 4.3
**6**	67.0 ± 3.7	71.0 ± 9.5
**7**	100.2 ± 6.0	99.5 ± 5.8
**8**	67.2 ± 3.0	65.2 ± 20.8
**9**	86.2 ± 12.8	-0.53 ± 1.5
**10**	97.7 ± 7.5	116.5 ± 41.4
**11**	1.38 ± 0.1	31.4 ± 11.9
**12**	92.6 ± 4.3	93.8 ± 1.4

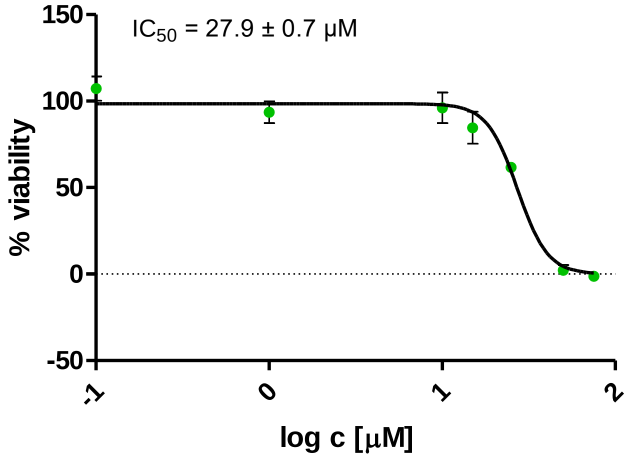

Figure S1. Dose-response curve for compound **11** in MTS assay in Hep G2 cell line, shown for an independent measurement in triplicate. The IC_{50} value (mean ± SD) is a result of three independent measurements.

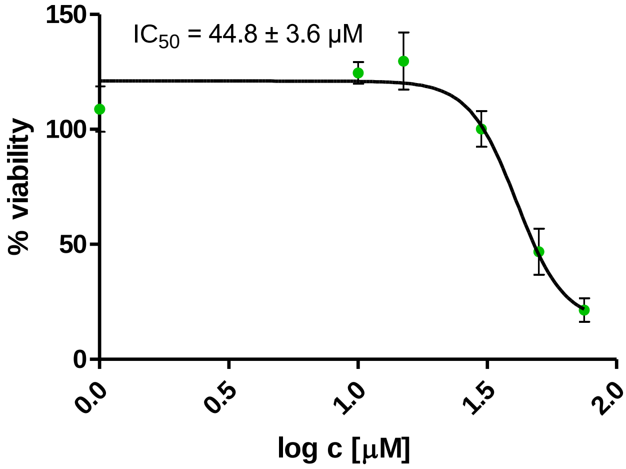

Figure S2. Dose-response curve for compound **11** in MTS assay in MCF-7 cell line, shown for an independent measurement in triplicate. The IC_{50} value (mean ± SD) is a result of three independent measurements.

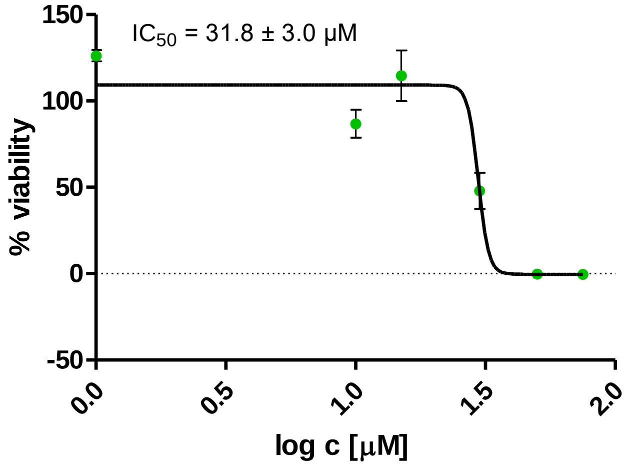

Figure S3. Dose-response curve for compound **9** in MTS assay in MCF-7 cell line, shown for an independent measurement in triplicate. The IC_{50} value (mean ± SD) is a result of three independent measurements.

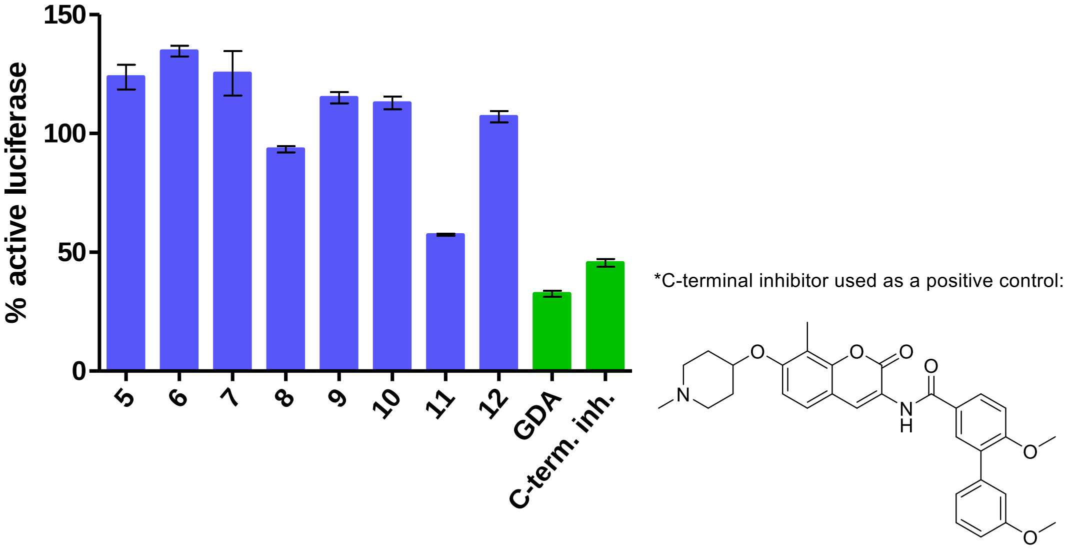

Figure S4. Luciferase refolding activity of Hsp90 in PC3 MM2 cells after treatment with compounds **5–12**, C-terminal inhibitor* and geldanamycin (GDA) at 50 μM concentration. Data are means ± SD of three independent experiments performed in triplicates.

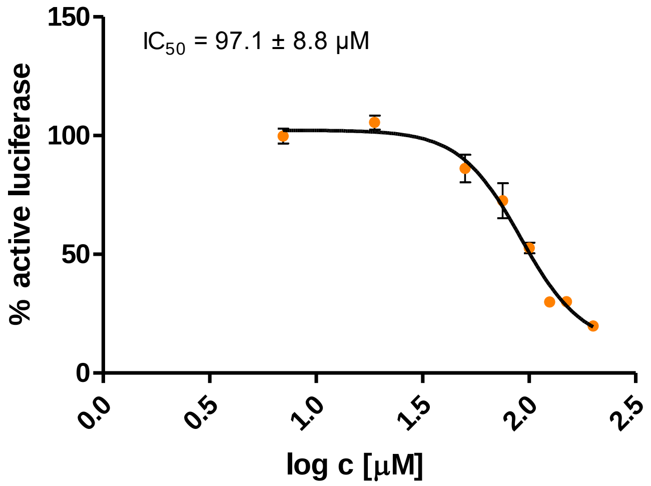

Figure S5. Dose-response curve for compound **11** in luciferase refolding assay on PC3 MM2 cell line, shown for an independent measurement in triplicate. The IC_{50} value (mean ± SD) is a result of three independent measurements.

References

1. Moroni, E.; Zhao, H.; Blagg, B.S.J.; Colombo, G. Exploiting Conformational Dynamics in Drug Discovery: Design of C-Terminal Inhibitors of Hsp90 with Improved Activities. *J. Chem. Inf. Mode.* **2014**, *54*, 195–208, doi:10.1021/ci4005767.
2. Garg, G.; Zhao, H.; Blagg, B.S.J. Design, Synthesis and Biological Evaluation of Alkylamino Biphenylamides as Hsp90 C-Terminal Inhibitors. *Bioorg. Med. Chem.* **2017**, *25*, 451–457, doi:10.1016/j.bmc.2016.11.030.
3. Davis, R.E.; Zhang, Z.; Blagg, B.S.J. A Scaffold Merging Approach to Hsp90 C-Terminal Inhibition: Synthesis and Evaluation of a Chimeric Library. *Med. Chem. Commun.* **2017**, *8*, 593–598, doi:10.1039/C6MD00377J.
